# Materia Medica and Therapeutics: An Introduction to the Rational Treatment of Disease

**Published:** 1891-06

**Authors:** 


*Materia Medica and Therapeutics: an Introduction to the Rational Treatment of Disease.* By J. MITCHELL BRUCE, M.D. Nineteenth Thousand. London: CASSELL & CO. LIMITED. 1891.

It is, we suppose, one of the mysteries of publishing into which we have no right to pry, that this issue of Dr. Mitchell Bruce's book is described as the "Nineteenth Thousand," instead of the Fourth Edition. There have been three previous editions, and the present preface is called the "Preface to this Edition." The principal changes introduced have been the accounts of the recent additions to the Pharmacopœia, and these have been effected without altering the number of pages. It is a book of exceeding merit, and one which we consider well-nigh indispensable to the practitioner.

